# The effect of nutritional education and continuous monitoring on clinical symptoms, knowledge, and quality of life in patients with cirrhosis 

**Published:** 2019

**Authors:** Pezhman Alavinejad, Eskandar Hajiani, Baharak Danyaee, Mehrnaz Morvaridi

**Affiliations:** *Research Center for Infectious Diseases of Digestive System, Ahvaz Jundishapur University of Medical Sciences, Ahvaz, Iran*

**Keywords:** Liver cirrhosis, Education, Nutrition, Booklet

## Abstract

**Aim:**

In this study, we aimed to evaluate the effectiveness of an educational intervention in patients with liver cirrhosis.

**Background:**

Liver cirrhosis is end stage of liver diseases with many complications that affects quality of life and nutritional status in cirrhotic patients. Today, educational intervention and nutritional counseling is a key factor for preventing disease process and improving quality of life in patients with chronic diseases.

**Methods:**

This quasi-experimental, non-randomized, pre and post intervention were conducted as a pilot study on cirrhotic patients. Educational intervention and nutritional counseling were performed in clinical visits and via guide booklet. Chronic liver disease questionnaire (CLDQ) and knowledge questionnaire were used to assess patients' quality of life and knowledge. Blood samples were taken before and after intervention for assessment of laboratory outcomes.

**Results:**

One hundred and seven patients referred to Research Center for enrolling in the trial. Twenty-eight did not meet inclusion criteria and seven patients were excluded from the study for lack of proper follow-up. Final analyses were done on 72 patients. Quality of life and knowledge of cirrhotic patients improved significantly after educational intervention (P < 0.0001). Biochemical characteristics were not changed significantly. Ascites and edema were improved significantly (P = 0.005 and P = 0.002, respectively). There was significant difference before and after intervention in the days of hospitalization (P = 0.001).

**Conclusion:**

We concluded a simple educational intervention and continuous monitoring for 6 months can affect clinical outcomes, quality of life, hospital admissions days, and knowledge of patients with cirrhosis.

## Introduction

 Liver cirrhosis is a chronic complicated disease and end stage of most liver diseases. Regeneration and fibrosis of hepatocytes that occurred in liver cirrhosis can lead to portal hypertension and synthetic dysfunction of liver (1-3). Today, the number of cirrhotic patients and deaths caused by advanced liver diseases are increasing. Annual death rate of cirrhosis is about 5 to 10 per 100,000 people worldwide (4). There are various causes for cirrhosis and chronic liver inflammation but the most commons include alcohol-related liver disease (ARLD), non-alcoholic fatty liver disease (NAFLD) and viral hepatitis. Based on severity, there are two types of cirrhosis, compensated and decompensated (5). Patients with liver cirrhosis are high risk group for developing hepatocellular carcinoma (HCC), irreversible complications of liver disease including varicose veins, and death due to liver disease (6). Therefore, continuous management of these patients is essential in early stages of disease for diagnosing HCC and esophageal varices (7). Ascites, variceal bleeding, and portal hypertension are the most common complications of liver cirrhosis (8). Malnutrition is a known complication in cirrhotic patients, and it seems that has significant prognostic effects on complications of disease such as liver encephalopathy, spontaneous bacterial peritonitis (SBP), hepatorenal syndrome, and ascites. This also leads to poor quality of life in patients (9). Almost all cirrhotic patients that are candidate for liver transplant suffer from some degree of malnutrition. Low calorie intake, due to appetite loss and suppression of hypothalamus by some hormones and cytokines such as cholecystokinin and tumor necrosis factor-alpha (TNF-alpha), is one of the most common causes of malnutrition in cirrhotic patients (10). In addition, alcohol can be another factor in reducing appetite in these patients. Other causes of decreased calorie intake and energy are presence of ascites, intestinal edema, and malabsorption (11). Knowledge and monitoring in cirrhotic patients is important because they need to take proper medications, adjust their lactulose dosage, and manage their diet. Therefore, successful management of hepatic cirrhosis requires education and continues monitoring for patients (12). Complexity and complications of liver cirrhosis restrict accessibility to information and understanding of disease process in patients. However, for long-term management, efforts to educate patients about etiology, pathology, and treatment of disease are essential (13). In fact, recent studies about patient education has been recognized as a key factor in supportive care of patients with liver cirrhosis (14). However, there is little information and evidence about the patient's knowledge about liver cirrhosis or the effectiveness of a routine educational intervention (7). Considering the importance of nutritional counseling, informing patients about their disease, improving quality of life, and reducing complications of cirrhotic patients, we aimed to conduct this study about the effectiveness of an educational intervention on laboratory tests, clinical symptoms, knowledge, and quality of life in patients with liver cirrhosis. 

## Methods

This clinical trial was confirmed in ethics committee of Ahvaz Jundishapur University of Medical Sciences (IR.AJUMS.REC.1396.811) and approved in Iranian Registry of Clinical Trials (IRCT20180625040237N1). The study protocol was conducted at Research Center for Infectious Diseases of Digestive System. 

**Participants**

Patients referred to Research Center for Infectious Diseases of Digestive System were included in trial with following criteria: 18 years and older, diagnosis of liver cirrhosis by gastroenterologist using ultrasonography, laboratory and clinical findings. Exclusion criteria were acute phase of liver cirrhosis, change in medications, malignancies, hepatorenal syndrome, severe heart and renal dysfunction, pregnancy or lactation, hepatocellular carcinoma, presence of infectious diseases and other cancers. After confirmation of inclusion criteria, patients filled consent form for enrolling in trial. We got informed consent from caregiver or family member in patients with encephalopathy.

**Design**

This quasi-experimental, non-randomized, pre and post intervention were conducted as a pilot study on patients referred to Research Center for Infectious Diseases of Digestive System. We requested from gastroenterologists for referring cirrhotic patients to Research Center. Patients were referred for the educational program and the number of clinical visits were equal for each participant. Patients filled baseline characteristics, liver cirrhosis knowledge, and chronic liver disease questionnaires. Nutritionist, physician and nurses educated patients about their illness and its management through face-to-face interview during two-hour visits. These educational contents included: liver function, liver cirrhosis (pathology, complications, treatment, medications, and management strategies), nutrition in cirrhosis, dietary components, healthy lifestyle advices, and dietary recommendations in liver cirrhosis. At the end of visit, a guide booklet about educational contents of session, dietary recommendations, and food exchange list were given to patients. Nurses followed up patients via text messages and weekly phone calls and answered participants’ questions. Also, patients reported changes in clinical symptoms and medications or hospitalization (If they had been hospitalized) to nurses. As we know many other educational sources are available for patients since we asked from patients that acted just based on nutritional recommendations that were provided in guide booklet and educational program. After 6 months, at the end of intervention, patients were visited and they filled questionnaires of liver cirrhosis knowledge and chronic liver disease. 

**Guide booklet**

We provided a booklet as titled “Nutritional Guideline in liver Cirrhosis” for participants. The booklet was included five chapters about liver cirrhosis disease, the importance of nutrition and diet in liver cirrhosis, dietary components and food groups, healthy lifestyle recommendations, and meal planning for cirrhotic patients. In chapter one, we introduce liver cirrhosis disease, liver function, epidemiology of liver cirrhosis, etiology, pathophysiology, and complications. We focused on the importance of healthy nutrition and adherence to proper diet for cirrhotic patients in chapter two. Dietary components (energy, protein, carbohydrates, fats, vitamins, and minerals) and food groups (grains, meats, dairy, vegetables, and fruits) were introduced in chapter three, also we focused on some dietary components for example sodium, water, and alcohol that have intake limitation for preventing complications of disease. In chapter four, we explained about healthy lifestyle recommendations such as nutritional management in obese cirrhotic patients, physical activity, food preparation methods, and healthy food pyramid. We provided some examples of meal planning (breakfast, lunch, dinner, and snacks) contains foods that leads to alleviate the complication of disease and medications in last chapter of the booklet. We explained about the educational content of guide booklet in clinical visits and used Images, food pyramid, and food exchange list for better understanding. 

**Assessment of quality of life and knowledge about disease**

In this study, chronic liver disease questionnaire (CLDQ) was used to assess patients' quality of life (15, 16). The questionnaire consists of 29 questions in 6 sections: emotional status (8 questions), abdominal symptoms (3 questions), physical activity (3 questions), systemic symptoms (5 questions), fatigue symptoms (5 questions), concern and anxiety (5 questions). Questions were evaluated on a scale of 1 to 7; always: 1 point, most often: 2 points, relatively often: 3 points, sometimes: 4 points, rare: 5 points, very rarely: 6 points, not at all: 7 points. Final score of quality of life is obtained by calculating total score of each patient. Validity of this questionnaire has been confirmed in previous studies in Iran (Cronbach's alpha = 0.93 and correlation = 0.89) (17). In this study, reliability of questionnaire was 0.84 by calculating Cronbach's alpha. Liver cirrhosis knowledge questionnaire included 20 questions in 4 sections, section one: pathology and etiology of liver cirrhosis, section two: health issues in liver cirrhosis, section three: taking medications, section four: nutritional management in liver cirrhosis. Nurses asked questions and patients answered: yes, I know (1 point), when they were informed about it or no, I don not know (0 point) when they were not informed. The level of liver cirrhosis knowledge was obtained by calculating total score of each patient. Content validity of the questionnaire was evaluated under counseling and guidance of two expert hepatologists in management of liver cirrhosis. Reliability of the questionnaire was 0.86 by calculating Cronbach's alpha.

**Laboratory tests**

Blood samples were taken after evaluation of inclusion criteria at the beginning of study and the end of educational intervention, then were frozen at -70 ° Celsius Immediately. Biochemistry tests included blood urea nitrogen (BUN) (used kit from Thermo Fisher Scientific Co, Massachusetts, united states), Na, K, Cr, Ca, alkaline phosphatase (ALP), Bilirubin direct, and Bilirubin total (used kits from Parsazmoon Co, Karaj, Iran), hemoglobin (used kit Chromosystems Co, Gräfelfing, Germany), aspartate aminotransferase and alanine aminotransferase (used kit from Man Co, Tehran, Iran). Thyroid-stimulating hormone (TSH) and Alpha-fetoprotein (AFP) were measured by Enzyme-linked immunosorbent assay (ELISA) method using Autobio Diagnostic kit (Autobio Diagnostic Co, Zhengzhou, China) and Pishtazteb kit (Pishtazteb Co, Tehran, Iran) respectively. International Normalized Ratio (INR) was calculated using formula (PT patient/PT reference plasma).

**Assessment of clinical outcomes and the days of hospitalization**

Clinical symptoms included ascites, edema, variceal bleeding, and encephalopathy were assessed at the beginning of study and 6 months after educational intervention. Physician used ultrasonography for diagnosis ascites when fluid was less than 500 ml and physical examination when accumulated fluid in abdomen was more than 500 ml. Diagnosis of edema was performed based on Edema Scale (graded on a scale 1+ to 4+) (18). Endoscopic examination was used to diagnose variceal bleeding and medical exam was performed by physicians for checking mental and neurological symptoms of encephalopathy. Hepatic encephalopathy (HE) is characterized by confusion, sleep disorders, decreased level of consciousness, and other neuropsychological symptoms. Severity of HE is graded based on the West Haven Grading System in five categories: minimal (some abnormalities on psychometric test), mild (change in behavior, mild confusion, stuttering, sleep disorder), moderate (moderate confusion, lethargy), severe (insensibility, sleeping, incompatibility in speech), and coma. Patients with minimal and mild grades of HE were included in our study. Caregiver for example a family member participates with encephalopathic patient in program and we presented all educational contents for both of them. We got informed consent from caregiver or family member in patients with encephalopathy. The days of hospitalization are the period of time that patients have been hospitalized for liver cirrhosis complications. At the begging of study, we asked from patients about the days of hospitalization in past six months. Participants had to report the days of hospitalization during six months after the educational intervention. Then, we compared it during six months before and after the program.

**Statistical analysis**

We performed statistical analyses using the SPSS package version 22 (SPSS Inc, Chicago, Illinois, USA). Data were analyzed by descriptive statistics including frequency distribution tables, charts, and central tendency (mean) and dispersion (amplitude and standard deviation) indices. Wilcoxon signed-rank test was used for comparing the means of variables before and after intervention. We used Chi-square test for comparing qualitative variables. Probability value less than 0.05 was considered significant. 

## Results

**Figure 1:**
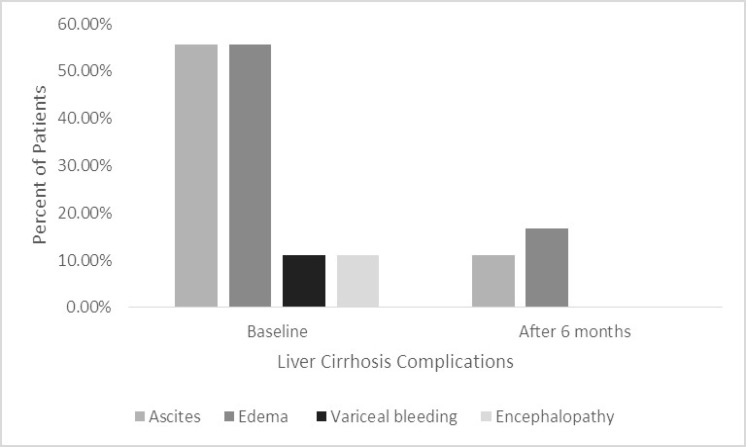
Clinical characteristics of subjects and outcomes after 6 months

One hundred and seven patients referred to Research Center for Infectious Diseases of Digestive System were evaluated for inclusion criteria. Twenty-eight did not meet inclusion criteria. The study was performed on 79 patients with liver cirrhosis. Since 7 patients were excluded from the study for lack of proper follow-up, all analyzes were done on remaining 72 patients. The average age of participants was 47/00 ± 17/42 years. Fifty six patients (77.8 %) were male and the educational status of participants was as follows: non-educated (11.1 %), under diploma (50.0 %), diploma (22.2 %), and upper diploma (16.7 %). Results of the study were divided into 5 categories: baseline, biochemical, clinical, quality of life, and knowledge. Baseline characteristics of participants are shown in Table 1. Biochemical characteristics at the beginning of study and after 6 months’ intervention were evaluated and reported in Table 2. There was no significant difference before and after educational intervention in biochemical characteristics of patients (P > 0.05). Clinical characteristics included ascites, edema, variceal bleeding, and encephalopathy. Percent of patients with ascites and edema decreased significantly after intervention, P = 0.005 and P = 0.002, respectively (Figure 1), but there was no significant difference in patients with encephalopathy (P = 0.157) and variceal bleeding (P = 0.157) before and after the study. The days of hospitalization decreased significantly before (1.28 ± 1.22 days) and after (0.33 ± 0.59) the program (P = 0.001). The difference of MELD score was not significant before (11.14 ± 3.59) and after (12.17 ± 4.84) intervention (P = 0.552). Quality of life was increased significantly after program (the scores before and after program were 4.22 ± 1.62 and 7.11 ± 0.83 respectively, P <0.0001). The knowledge scores improved significantly before (141.89 ± 20.40) and after (182.72 ± 10.27) educational intervention (P < 0.0001).

**Table 1:** Baseline characteristics of subjects

Variables	Mean±SD /percent	Variables	Mean±SD /percent
Weight (kg)	68.09 ± 16.04	Cause of liver cirrhosis HBV HCV AIH Fatty liver Idiopathic	8 (11.1 %) 32 (44.4 %) 12 (16.7 %) 4 (5.6 %) 16 (22.2 %)
Height (cm)	170.00 ± 10.49		
BMI (kg/cm^2^)	24.06 ± 5.07		
Marital status (Married)	60 (83.3 %)		
Alcohol consumption	8 (11.1 %)		
Smoking	24 (33.3 %)		
Ethics group Lur Arab Persian Others	20 (27.8 %) 32 (44.4 %) 12 (16.7 %) 8 (11.1 %)	Family history of diseases Liver Renal Diabetes Others	12 (16.7 %) 8 (11.1 %) 12 (16.7 %) 12 (16.7 %)

Data are expressed as Mean±SD or percent; Abbreviations: BMI: Body Mass Index, HBV: Hepatitis B Virus, HCV: Hepatitis C Virus, AIH: Autoimmune Hepatitis

**Table 2:** Baseline biochemical characteristics of subjects and outcomes after 6 months

Variables	Baseline	After 6 months	P-value	Variables	Baseline	After 6 months	P-value
BUN (mg/dL)	16.12 ± 10.50	17.48 ± 8.11	0.39	ALT (IU/L)	52.11 ± 29.96	43.46 ± 20.40	0.47
Cr (mg/dL)	0.93 ± 0.37	1.09 ± 0.36	0.16	AST (IU/L)	64.72 ± 36.45	53.92 ± 18.68	0.39
Na (mmol/L)	138.71± 2.80	139.58 ± 2.71	0.24	ALP (IU/L)	311.44 ± 215.50	271.50 ±138.35	0.83
K (mg/dL)	4.02 ± 0.38	4.11 ± 0.35	0.13	INR	1.34 ± 0.42	1.29 ± 0.29	0.65
Hb (g/dL)	11.73 ± 2.36	11.47 ± 1.41	0.34	TSH (µg/ml)	2.92 ± 4.51	3.85 ± 4.14	0.89
Ca (mg/dL)	8.84 ± 0.86	9.08 ± 0.24	0.85	Bil (total) (mg/dL)	1.89 ± 1.33	1.60 ± 0.83	0.13
AFP (µg/L)	8.16 ± 10.45	7.61 ± 11.33	0.73	Bil (direct) (mg/dL)	0.59 ± 0.58	0.61 ± 0.35	0.32

Data are expressed as Mean±SD; Abbreviations: BUN: Blood Urea Nitrogen; Cr: Creatinine; Hb: Hemoglobin; AFP: Alpha-fetoprotein; ALP: Alkaline phosphatase; AST: Aspartate transaminase; ALP: Alkaline Phosphatase; INR: International Normalized Ratio; TSH: Thyroid-Stimulating Hormone; BILL: Bilirubin.

## Discussion

Results of our study have shown that an educational intervention along with regular treatment was effective in cirrhotic patients for improving quality of life, knowledge, complications of disease including ascites, edema, and the days of hospitalization. Some studies evaluated the effect of knowledge and education in cirrhosis management, although in our study educational program focused on nutritional approaches. To the best of our knowledge this is first study about the impact of nutritional education on laboratory and clinical outcomes. Wigg *et al.* investigated the effectiveness of a chronic disease management model (CDM) in patients with liver cirrhosis. The results showed that despite the improvement in severity of disease (based on MELD and child scores) and all aspects of quality of life (based on CLDQ score) at the end of follow-up period, the intervention had no significant effect on these variables (19). In our study, educational intervention had significant effect on ascites, edema, quality of life, knowledge, and the days of hospitalization. One reason for the differences is the type of intervention. In this study, CDM included: delivery support, self-management support, decision support, and clinical information, while in our study, information focused on role of nutrition in management of disease and nutritional care such as restriction of salt consumption and foods with high sodium content which can lead to exacerbation of ascites and edema. In a study by Volk *et al*., knowledge of self-management in cirrhotic patients was evaluated. They reported that patient knowledge improved significantly with simple educational intervention (using a brief booklet) compared to the beginning of study. In this study, guide booklet provided nutritional and non-nutritional information (12). In other study, Beg *et al.* determined the impact of information leaflet on level of knowledge in cirrhotic patients. The educational content of leaflet was about understanding liver cirrhosis, complications, surveillance, salt and alcohol intake, and medications. Results suggested that leaflet improved understanding of disease significantly (20). The results of our study also showed that patients’ knowledge increased significantly after educational intervention with using guide booklet (P-value < 0.0001). Unlike other studies, the educational content of sessions and guide booklet focused on nutritional recommendations and meal planning. Goldsworthy *et al.* reported that patient knowledge about liver cirrhosis was weak at the beginning of study and significantly improved after multimedia education. Therefore, the educational intervention in this study was an effective way for management of liver cirrhosis (7). These results are matched with findings of the present study but the educational tool was different. Kadokawa *et al.,* in a study, examined the effectiveness of educational classes for patients with chronic liver diseases. The knowledge level of patients and their families improved significantly after attending in classes and recovery rate was dependent on the number of attendance in the classes. Educational content of these classes was about prophylaxis and treatment of hepatic cancer, iron restriction, and effect of branched-chain amino acids (21). In our study patients' knowledge about liver cirrhosis was weak at the beginning of the study and after intervention, the level of knowledge increased significantly. Physicians in clinical visits usually don not inform patients about nutritional approaches in treatment of cirrhosis and patients don not care about their nutritional status. Because of novelty of the nutritional recommendations that were interesting for patients in our study, they affected motivation, participation and learning of patients in clinical visits. Therefore, further studies are needed to determine the factors affecting patient knowledge and effectiveness of educational intervention. Overall, the results of this study showed that educational intervention improved patient knowledge significantly and can be used as a useful method to increase the awareness of patients with liver cirrhosis in clinical setting. In a study, Zandi *et al*. reported that self-care educational program and continuous monitoring in patients with cirrhosis for three months increased quality of life significantly (17). In our study also the quality of life of patients after intervention increased significantly (P-value < 0.0001). Other studies have shown that protein-energy malnutrition is associated with reduction in survival of patients with liver cirrhosis (22, 23). Nutritional status is essential in managing patients with advanced hepatic diseases. Malnutrition in cirrhotic patients increase risk of morbidity and reduce quality of life due to adverse side effects (24). Generally, the results of this study showed that the intervention had positive effects on quality of life in patients with liver cirrhosis. By improving quality of life, their attitude to treatment can be more promising and have better effects. In the present study, laboratory parameters and MELD score were not significantly altered at the end of intervention. One of the reasons is duration and personalization of educational intervention. Probably significant changes in laboratory characteristics need long term and personalized intervention according to disease history of patients including pattern of changes in laboratory parameters and design nutritional education for each patient. The other reason is the progressive nature of liver cirrhosis that affect laboratory parameters and MELD score in all stages of disease. Since the prevalence of chronic liver diseases is increasing, the intervention in this study can be used to make empirical evidence in this area such as the impact of educational intervention, continues monitoring, and nutritional care for development of some factors such as participation in HCC screening, adherence to treatment, and health outcomes. It should be noted that the measurement and improvement of understanding and knowledge of patients about disease and treatment is difficult and may be influenced by several confounding factors such as health literacy, beliefs, health status, medications, and treatment, relationship and interaction of patient-physician (such as the quality of education and communication), self-efficacy, and the impact of other internal and external barriers (25-27). Therefore, interventions in this area should be included various and wide range of patients. Physicians and nurses usually ignore education as a part of treatment process while it is an important component that impact on self-care activities and self-monitoring. Active participation of patients and their families in treatment program can lead to achieve better understanding about the disease and help for improving quality of treatment. Education about the disease encourage patients to continue their treatment and have reasonable expectations from treatment results. The results of our study showed that a simple educational intervention and continuous monitoring for 6 months can affect clinical outcomes, quality of life, hospital admissions days, and knowledge of patients with cirrhosis. Main limitation in our study was the method of evaluation for knowledge that depended on self-assessment and we did not have any objective measurement. Other limitations include, lack of long term follow-up and control group, and single-centered study that allowed the participation of a specific group of patients. Our suggestions for future studies are performing interventions on large number of patients, longer period time of intervention, increase the number of educational sessions and clinical visits, design a randomized controlled trial for comparing results between two groups, and providing educational sessions for patients’ families and their nurses.
